# Pregnancy-induced obsessive compulsive disorder: a case report

**DOI:** 10.1186/1744-859X-4-12

**Published:** 2005-06-15

**Authors:** Harish Kalra, Rajul Tandon, Jitendra kumar Trivedi, Aleksandar Janca

**Affiliations:** 1 Department of Psychiatry, Royal Perth Hospital, Perth WA 6000, Australia; 2 Grampians Psychiatric Services, Ballarat Health Services, Ballarat VIC 3350, Australia; 3 Department of Psychiatry, King George Medical University, Lucknow – 226003, U.P., India; 4 University of Western Australia, School of Psychiatry and Clinical Neurosciences, Perth WA 6000, Australia

## Abstract

Pregnancy is a well-recognised risk factor in precipitating obsessive-compulsive disorder. We present and discuss a case with the onset of obsessive-compulsive disorder in the fourth month of gestation, which fully recovered two weeks after delivery. The phenomenology of the observed disorder was similar to earlier reports of obsessive-compulsive disorder in pregnancy, i.e. the obsessions and compulsions were predominantly related to the concern of contaminating the foetus resulting in washing compulsions. Despite the initial success with anti-obsessional drugs, the patient stopped the medication in the last month of gestation. Nevertheless, she fully recovered two weeks after the delivery without any psychiatric intervention. There were no obsessive-compulsive symptoms at one-year follow up. The possible mechanisms involved in the aetiology of this case, and future research directions in understanding the role of pregnancy in OCD are discussed.

## Introduction

Pregnancy and the postpartum period are known to influence the onset and course of various psychiatric disorders such as mood disorders, psychotic disorders, and anxiety disorders. [1-3] There is considerable evidence that suggests the role of stressful events, including pregnancy and childbirth, in precipitating or exacerbating obsessive-compulsive disorder (OCD). [4] Various studies have evaluated the role of pregnancy in OCD and have reported onset and exacerbation of OCD in a significant percentage of their study groups. [5-7]

Postpartum OCD has been described as having onset within the first three weeks of delivery. [8,9] Here we report a patient whose OCD had its onset during pregnancy and remitted following delivery. To the best of our knowledge, this is the first report describing onset of OCD during pregnancy with spontaneous complete recovery following delivery.

## Case presentation

A 30-year old primigravida woman presented to the outpatient department in the fourth month of gestation. She had no past history of psychiatric illness. Her chief complaints were contamination obsessions and washing compulsions in the preceding one month. Preoccupied with thoughts of contamination, she had started spending the majority of time washing herself or cleaning various household items. She described these thoughts as being her own and recognised them to be "irrational", but she could not resist them. She was distressed and unable to maintain her employment. Washing compulsions relieved her anxiety. However, she could not offer an explanation as to what she feared about contamination. No depressive or psychotic symptoms were elicited. Biochemical investigations, including metabolic and thyroid function studies, were in the normal range. She was diagnosed with OCD according to ICD-10 criteria [10].

Pharmacotherapy, offered at the first consultation, was refused by the patient because of her (non-obsessional) concerns about teratogenic effects of drugs. Behavioural therapy in the form of thought stopping was begun. The patient reported exacerbation of symptoms at the next consultation and subsequently disclosed that her obsessional thoughts also concerned the fear of contaminating her unborn baby. She repeatedly washed to avoid damage to her foetus. One month after her initial presentation, fluoxetine was started, at an initial dose of 20 mg/day gradually and gradually increased to 60 mg/day over the next four weeks. The patient reported reduction of obsessional and compulsive symptoms, and was able to resume her work. She remained on fluoxetine until the eighth month of pregnancy with no reports of exacerbation. However, the patient stopped the medication during the last month of gestation on the alleged advice of family members. The patient again experienced the relapse of intrusive obsessional thoughts followed by compulsions, but refused to resume pharmacotherapy until delivery. The patient returned to the outpatient clinic fifteen days postpartum. The patient described no obsessive thoughts or washing compulsions for the preceding one week. She was followed up for five visits in the next one year without any reports of obsessions or compulsions.

## Discussion

There is evidence supporting the role of major life events including pregnancy and delivery in precipitating OCD. [4] However, to the best of our knowledge, there are no specific reports showing complete resolution of OCD after delivery in cases having onset during pregnancy. In two studies addressing the role of pregnancy in OCD, Neziroglu et al [5] and Williams et al [6] found pregnancy to be associated with onset of OCD in 39% and 13% patients, respectively. It occurred in primigravida in 52% of the patients. [5] Our case too had its onset in her first pregnancy at the fourth month of gestation. Our patient had major symptoms in the form of obsessions of contamination and compulsions with the underlying fear of contaminating her foetus. This phenomenology is in consonance with the literature. [4,11] Purely intrusive obsessional thoughts with the same underlying theme have also been described in a case series of postpartum OCD. [8]

The underlying mechanism can only be speculative at this stage. As full recovery was seen after delivery, our case report negates the proposed mechanism in postpartum OCD of adverse impact on serotonergic functions by rapid withdrawal of oestrogen and progesterone in postpartum period [9]. We propose it to be considered as equivalent to chorea gravidarum, which is also characterised by onset of involuntary movements during pregnancy with complete resolution after delivery. [12] Basal ganglia abnormalities are known to occur in pregnancy as in chorea gravidarum. Similarly, basal ganglia pathology, especially involving the caudate nucleus, has been implicated in OCD [13-15]. We hypothesize that similar mechanisms may underlie both chorea gravidarum and this case of pregnancy-induced OCD.

An underlying mechanism proposed for chorea gravidarum of enhanced dopaminergicsensitivity under the effect of elevated levels of female sex hormones due to pregnancy [16] could also be presumed as operating in this case but involving serotonin instead of dopamine. Our patient showed significant improvement with fluoxetine before being ceased by the patient, thus indirectly supporting serotonin dysfunction. Previous reports of post-partum OCD have also shown good response to fluoxetine. [9]

Careful prospective studies of pregnancy-associated OCD will help in understanding predisposing and aetiological factors involved in such cases. Comparison of chorea gravidarum and OCD in pregnancy by functional imaging techniques like PET/SPECT/fMRI might prove useful in understanding pathophysiological processes responsible for these disorders.

## Competing interests

The author(s) declare that they have no competing interests.
